# Memoranda of a Supposed Case of Ruptured Uterus Consequent upon a Partial Adhesion of the Vaginal Parietes

**Published:** 1829

**Authors:** Richard F. Barrett

**Affiliations:** Greensburg, Kentucky


Art. III.—*Memoranda of a supposed case of Ruptured Uterus consequent upon a Partial Adhesion of the Vaginal Parietes.* By Richard F. Barrett, M. D. of Greensburgh, Kentucky.

The subject of the following case, Mrs. S. of Barren county, in this state, died in the manner I am about to describe, in her 29th year, having been married 11 years.

During the first year of her marriage, she suffered two abortions; the first in the third month of pregnancy, and the second in the fifth or sixth. There were no unusual symptoms attending the first; but in the second, there was excessive hæmorrhage, and a retention of the placenta, for the extraction of which many forcible efforts were unsuccessfully made. These efforts, however, had the unfortunate effect of prolapsing the uterus, and of injuring the vagina so much, as to give rise to inflammation and sloughing of its coats. From unskilful management during the healing process, the walls of the organ were suffered to unite; and thus was formed the obstruction which ultimately occasioned death.

From the time of her second abortion to her last confinement, a period of eleven years, her health was variable. At length she again became pregnant, and on Tuesday night, Sept. 30th, 1828, was taken in labor. The pains were regular and moderate until Friday night, the 10th of October, at which time the liquor amnii was discharged. The head of the fœtus now could be distinctly felt resting on the abnormal vaginal septum. The pains after this increased in violence, and for twenty-four hours were so strong that the attendants thought at every returning throe the obstruction would be overcome, and the child delivered. About 12 o'clock the ensuing night, Oct. 11th, the good old ladies who surrounded her, unfortunately determined to expedite the event by rolling the patient over and over on the floor, and then turning her feet *upwards*, and shaking her violently. This agitation, together with the strong uterine action that existed at the time, had, most probably, the unfortunate effect of rupturing the uterus; for immediately afterwards the head of the child could not be felt resting on the septum, the uterine pains ceased, hæmorrhage occurred, with sickness, and a sudden sinking of the powers of life. From this time until Monday the 13th, her friends expected her to die every hour; but at that time finding her better, or at least no worse, they determined to send to this place for my partner, Dr. Taylor. He reached the house that evening, and found her pulseless at the wrist, with cold extremities, and other symptoms indicating the near approach of death. Stimulants were administered through the night; but the next morning there was little or no amendment in her condition. Business requiring his attention at home, he was compelled to leave her, but directed a continuation of the stimulant plan, and in case there was a return of uterine action, or an increase of the powers of life, requested the husband of the lady to send for him and myself, promising that we would visit her, and either cut through the vaginal obstruction, or perform the Cæsarian section. On Thursday, the 16th, there was a return of circulation to the extremities, and a general warmth of the system, with wandering pains of the abdomen, back, hips, and thighs. These symptoms continuing through the following day, the friends felt encouraged, and accordingly sent for us. We found her undelivered, and evidently in the last hours of existence. She had been in labor nineteen days. She was in a clammy sweat, with cold extremities, a sunken, anxious countenance, hiccough, and a pulse just perceptible at the wrist. Believing, from the history of her labor, as given by the attendants, that a rupture of the uterus had taken place several days before, that the child was dead, and that from the existing symptoms she must inevitably die in a few hours, we of course declined all surgical assistance. On examination *per vaginam*, the cause that had prevented delivery, was immediately ascertained. About an inch, or an inch and a half, within the vulva, we discovered that a firm adhesion of the anterior and lateral walls of the vagina had taken place, leaving only an aperture of one or two lines in diameter, near the recto-vaginal septum. The exact longitudinal extent of the adhesion, could not be correctly ascertained; but from examination with the finger and probe, we judged it to be five or six lines.

Convinced that no human power could prevent the fatal termination of the case, and other professional business imperiously demanding our attention at home, we left the unfortunate woman before dissolution. We learned, however, that she died eight or ten hours afterwards, nineteen days after the commencement of her labor and seven days after the uterus appears to have been ruptured.

Residing 18 miles from the patient, we could not return to make a *post mortem* examination, and ascertain whether a rupture of the uterus had actually taken place, as well as the extent of the adhesion. But from the recession of the head of the fœtus from the vagina, the cessation of uterine action, the nausea, the hæmorrhage, and sinking of the vital powers, which immediately followed the severe rolling and shaking of the patient, we felt scarcely a doubt of the fact.

*September 2d*, 1829.

### Editorial Note.

We cannot but regret with our friend and correspondent, that a dissection had not been made in this case. It would either have verified or falsified the prognosis of a rupture of the uterus. If it occurred at the time supposed, the continuance of the life of the patient for seven days afterwards, might be regarded as a remarkable circumstance. Under the supposition of its existence, we have thought the case worthy of being laid before the profession. It is deserving of publication, moreover, as a melancholy example of reckless and ignorant officiousness, on the part of the original attendants. The violence which led to an inflammation and coalitition of the sides of the vagina, must have been rude in the extreme; and yet it was surpassed by the outrages which seem finally to have lacerated the uterus. On the circumstances under which impregnation took place, the physiologist may perhaps reflect with some advantage.

